# Stability and volatility shape the gut bacteriome and *Kazachstania slooffiae* dynamics in preweaning, nursery and adult pigs

**DOI:** 10.1038/s41598-022-19093-9

**Published:** 2022-09-05

**Authors:** Brandi Feehan, Qinghong Ran, Victoria Dorman, Kourtney Rumback, Sophia Pogranichniy, Kaitlyn Ward, Robert Goodband, Megan C. Niederwerder, Katie Lynn Summers, Sonny T. M. Lee

**Affiliations:** 1 Division of Biology, College of Arts and Sciences, Kansas State University, Manhattan, KS 66506 USA; 2 Department of Animal Sciences and Industry, College of Agriculture, Kansas State University, Manhattan, KS 66506 USA; 3 Department of Diagnostic Medicine/Pathobiology, College of Veterinary Medicine, Kansas State University, Manhattan, KS 66506 USA; 4 Animal Biosciences and Biotechnology Laboratory, Beltsville Agricultural Research Center, Agricultural Research Center, United States Department of Agriculture, Beltsville, MD 20705 USA; 5 Swine Health Information Center, Ames, IA 50010 USA

**Keywords:** Computational biology and bioinformatics, Microbiology

## Abstract

The gut microbiome plays important roles in the maintenance of health and pathogenesis of diseases in the growing host. In order to fully comprehend the interplay of the gut microbiome and host, a foundational understanding of longitudinal microbiome, including bacteria and fungi, development is necessary. In this study, we evaluated enteric microbiome and host dynamics throughout the lifetime of commercial swine. We collected a total of 234 fecal samples from ten pigs across 31 time points in three developmental stages (5 preweaning, 15 nursery, and 11 growth adult). We then performed 16S rRNA gene amplicon sequencing for bacterial profiles and qPCR for the fungus *Kazachstania slooffiae*. We identified distinct bacteriome clustering according to the host developmental stage, with the preweaning stage exhibiting low bacterial diversity and high volatility amongst samples. We further identified clusters of bacteria that were considered core, increasing, decreasing or stage-associated throughout the host lifetime. *Kazachstania slooffiae* was absent in the preweaning stage but peaked during the nursery stage of the host. We determined that all host growth stages contained negative correlations between *K. slooffiae* and bacterial genera, with only the growth adult stage containing positive correlates. Our stage-associated bacteriome results suggested the neonate contained a volatile gut microbiome. Upon weaning, the microbiome became relatively established with comparatively fewer perturbations in microbiome composition. Differential analysis indicated bacteria might play distinct stage-associated roles in metabolism and pathogenesis. The lack of positive correlates and shared *K. slooffiae*-bacteria interactions between stages warranted future research into the interactions amongst these kingdoms for host health. This research is foundational for understanding how bacteria and fungi develop singularly, as well as within a complex ecosystem in the host’s gut environment.

## Introduction

Host-associated microbiomes have critical roles in host health, growth and development. The digestive system contains microbes with a wide array of functions for hosts, such as aiding in nutrient availability, protecting from pathogen invasion and maintaining a healthy gut epithelial barrier^1–3^. An imbalance of microorganisms, or their associated functions, in this enteric, or digestive, microbiome can lead to a dysbiotic state and diseased host^1^. Diseases and symptoms associated with a dysbiotic enteric microbiome include inflammatory bowel disease (IBD), diarrhea, obesity, and metabolic syndrome (MetS), among other ailments^4^. In order to develop therapies for these illnesses, it is paramount to understand the enteric microbiome dynamics spanning microbial kingdoms, including bacteria and fungi, throughout the lifetime of swine hosts.

Foundational to evaluating microbial interactions is first determining dynamics of the gut during the host lifetime. Traditionally, scientists have evaluated microbial composition and alpha (⍺) diversity to understand gut microbial development^5^. Composition includes the overall taxonomic comparison amongst samples whereas ⍺ diversity quantifies how many distinct taxa are present. During the host lifetime, we expect to see compositional similarity in similar aged hosts, but distinctions over time as microbes change in abundance^6–8^. Contrastingly, we expect to see ⍺ diversity develop in the early lifetime until the gut environment reaches a relatively staple level of development amongst hosts^1,2^. A developed gut is expected to have a relatively higher ⍺ diversity, compared to a developing environment, indicating a diverse microbial makeup with similarly diverse roles within the microbiome system and for host health^9,10^. By evaluating the microbial composition and ⍺ diversity we will have a foundational understanding of how the gut microbiome develops during the host lifetime which aids investigations into bacterial and fungal interactions.

As mentioned previously, understanding microbial correlations and interactions between microbial kingdoms, including *Fungi* and *Bacteria*, are critical to elucidating diseases impacted by these microbial kingdoms. Previous research has shown a negative correlation, indicating a competitive relationship, between bacterial diversity and fungal abundance^11^. Still, the microbial mechanisms, influencing other microbes and the host alike, underlying these outcomes have not been described. We must understand bacterial-fungal interaction intricacies to provide treatments targeting specific microbes and mechanisms, especially those of bacterial-fungal dysbiotic gut microbiomes.

Current research lacks an understanding of how the dominant swine enteric fungus, *Kazachstania slooffiae*, changes in the majority of the swine lifetime, and how these changes are influenced by the bacterial communities. *Kazachstania slooffiae* is a member of the *Saccharomycetaceae* family, and the fungus is a proposed commensal in the swine gut microbiome^12^. Studies indicate *K. slooffiae* dominates the mycobiome from 70 to 90% of total yeasts, especially following weaning^13,14^. The fungus has been demonstrated to significantly alter the gut microbiota during weaning, leading to a potentially beneficial increase in short chain fatty acid (SCFA) concentration^15^. Although *K. slooffiae* is the primary fungus after weaning, we currently lack a longitudinal understanding of this fungus. Publications have only evaluated *K. slooffiae* abundance from birth until 39 days of age^13,16^. The average time to market age is 160 days, so prior publications have only evaluated *K. slooffiae* dynamics in 25% of the swine lifetime to market^17^. Moreover, previous studies have identified eight SparCC correlations between *K. slooffiae* and bacterial genera from nursery-aged hosts^18,19^. We hypothesized there were more inter-kingdom correlations occurring throughout the swine lifetime (including preweaning and growth adult as these were not studied previously) which influence microbiome establishment and host health^11^. Our study aimed to elucidate novel stage-associated bacteriome-*K. slooffiae* correlations to build a foundation for future inter-kingdom interaction studies.

This study highlights development of bacteria, fungi and host, with an investigation into bacterial-fungal correlations. We followed ten swine from birth. We first determined the foundational gut microbiome development during the host lifetime. Studies have demonstrated various factors from biology, such as host diet and housing environment, to methodology, including DNA extraction and bioinformatic approaches, impacting resulting identification of microbes and microbial diversity^20–22^. Therefore, we aimed to first provide a baseline understanding of how our gut bacteria changed in the lifetime of the ten swine hosts. With this knowledge, we could then further interpret how the microbial composition and ⍺ diversity pertained to microbial inter-kingdom interactions and potential implications on host health at various ages. Understanding inter-kingdom interaction, influenced by gut development, in the swine may provide insights into the intricate relationship between the host and the microbiome. Foundation to this longitudinal study, swine were grown in three stages which varied according to host development, diet and housing: preweaning (milk diet and housed with littermates and dam; birth-21 days of age), nursery (pellet diet and co-housed with other litters; 21–80 days) and growth adult (pellet diet and co-housed with other litters; 80–122 days). As discussed previously, directly following weaning into the nursery stage in swine hosts, one fungus has been consistently identified in the enteric mycobiome: *Kazachstania slooffiae*^14,19^. For this reason, our study focused on elucidating longitudinal dynamics between *K. slooffiae* and bacteria.

In our study, we determined specific host-age and -dietary stage microbiome development characteristics. These included an increasing microbial diversity, decreasing volatility and increasing fungus *K. slooffiae* in the young host (preweaning and nursery developmental stages). The older host (growth adult stage) microbiome was relatively established with a complex correlation network amongst bacteria and *K. slooffiae*. Together, these findings indicated a dynamic microbiome development from birth until weaning with an increasing number of inter-kingdom interactions throughout the host lifetime.

## Materials and methods

### Hosts and study design

We followed ten swine over the course of their lifetime, with fecal collections, rectal temperature, weights, and general health observations collected from 2 to 157 days of age, to understand successive shifts in microbial populations (Supplemental Table S1; Supplemental Document S1). We started the study with ten swine, but one pig died prematurely at 28 days of age. The experimental unit was each individual swine. The hosts were housed indoors and fed distinct diets according to their stage of life. Five dams were randomly selected from the same farrowing group, and one male and one female were randomly selected per dam. Swine were housed with their dam in the preweaning stage, in groups of five in the nursery stage, and all in one pen during the growth adult stage. Hosts were sampled in three stages: preweaning, nursery, and growth adult. The preweaning diet consisted of mother’s milk and potentially feed as the hosts grew old enough to reach their mother’s trough. Nursery diet, phase 1, transitioned from milk to pelleted feed after weaning from the mother and moving into a new barn environment. A second pelleted feed was fed during nursery phase 2, while a meal was fed for nursery phase 3. The growth adult stage also included three phase diets with an initial move into another barn environment accompanying the nursery-growth transition. Hosts did not receive antibiotics or antifungals prior to or during the study. Males were castrated during the preweaning stage. Pigs were managed according to the Kansas State University Institutional Animal Care and Use Committee (IACUC) approved protocol #4036, and methods are reported according to ARRIVE guidelines. Additionally, the authors confirmed that all methods were performed in accordance with relevant guidelines and regulations, and all methods were approved by Kansas State University.

We performed fecal collection with a fresh set of sterile gloves using the free-catch method, prior to contact with the ground. We collected fecal samples every 5 days during preweaning and nursery stages, and every seven days during the growth adult stage. Immediately after collection, samples were stored in either a sterile 15 mL tube or sterile bag, kept on ice, and then transported to the laboratory for subsequent storage at −80 °C until genomic DNA extraction.

### DNA extraction and marker gene sequencing

We used the E.Z.N.A.^®^ Stool DNA Kit (Omega Bio-tek, Inc.; Norcross, GA) to extract the microbial DNA from the fecal samples. We used the manufacturer pathogen detection protocol without bead beating and utilized only 30 μL elution buffer per sample. Extracted DNA was quantified with Nanodrop and a Qubit™ dsDNA BR Assay Kit (Thermo Fisher; Waltham, MA) for sample DNA quality and concentration. DI water was utilized during quantification as a negative control. Extracted microbial DNA was stored at −80 °C until library preparation. Bacterial 16S rRNA gene V4 region was amplified during library preparation via Illumina’s Nextera XT Index Kit v2 (Illumina, Inc.; San Diego, CA) (primers: 515F, GTGCCAGCMGCCGCGGTAA and 806R, GGACTACHVGGGTWTCTAAT)^23^. Library preparation and subsequent sequencing also included a no template negative control. Sequencing was done on an Illumina MiSeq which generated paired-end 250 bp reads.

### Kazachstania slooffiae qPCR

We performed the *K. slooffiae* qPCR, with the SensiMix™ SYBR^®^ Hi-ROX Kit (Bioline, Meridian Bioscience; Cincinnati, OH), as previously described (primers: KS-f, ATCCGGAGGAATGTGGCTTC and KS-r, AGCATCCTTGACTTGCGTCG)^13^. Master mix components and qPCR conditions are listed in Supplemental Table S2. Each qPCR run included at least one PCR-grade water with the master mix as a non-template control (NTC), with one *K. slooffiae* positive sample repeatedly used across plates as the positive control.

### Bioinformatic and statistical analysis

We used cutadapt and DADA2 in QIIME2 v2019.7 (https://qiime2.org/) to trim and perform quality control for the sequencing reads (Supplemental Table S3)^24,25^. Reads in which no primer was found were discarded. The reads were truncated at locations where 25-percentile of the reads had a quality score below 15. Diversity analysis was carried out at a sampling depth of 11,105 reads. The pre-trained classifier offered by QIIME2 using the SILVA version132 (https://www.arb-silva.de/documentation/release-132/) database was used for taxonomic assignment of bacteria^26–28^. We used a weighted UniFrac, generated from QIIME2, on the rarefied dataset (11,105 reads) to evaluate differential microbial composition among the samples in different stages, and we utilized a QIIME2 principal coordinate analysis (PCoA) to visualize the microbial composition structure based annotated ASVs (Supplemental Document S2). The following applications were utilized in generating the PCoA composition plot with RStudio version 1.3.1093 (https://www.rstudio.com/products/rstudio/older-versions/): tidyverse version 1.3.1 (https://cran.r-project.org/package=tidyverse), qiime2R version 0.99.6 (https://github.com/jbisanz/qiime2R), plyr version 1.8.7 (https://www.rdocumentation.org/packages/plyr/versions/1.8.7), and ggpubr version 0.4.0 (https://CRAN.R-project.org/package=ggpubr)^29–33^. We calculated ⍺ diversity to represent the species diversity in each sample. We utilized Shannon index, estimated number of species (ENS), and Faith’s phylogenetic diversity, all within QIIME2, to measure the number of ASVs and the uniformity of ASV abundance for diversity evaluation (Supplemental Document S2)^25^. Kruskal–Wallis was used in QIIME2 to provide overall and stage pairwise statistical analyses for Shannon, ENS, and Faith’s phylogenetic diversity^25^. We calculated Shannon effective number by calculating the exponential [exp(H)] of the original Shannon diversity index (H)^34^. We used PERMANOVA in QIIME2 on Bray–Curtis dissimilarity index to test if there were statistically significant differences between stages^25^. Volatility results were analyzed within QIIME2 on the first axis of the PCoA to indicate how dispersed samples were at the associated swine age^25^.

We further used DESeq2 version 1.30.1 (https://github.com/mikelove/DESeq2), in RStudio, to mark the statistical differences in the bacterial populations (phyla and genera) predominance between the stages and to generate heatmaps with pheatmap version 1.0.12 (https://CRAN.R-project.org/package=pheatmap)^33,35,36^. Adjusted p-values were utilized, rather than standard p-values, as the adjusted values incorporated the Benjamini–Hochberg false discovery rate (FDR)^35,37^.

16S rRNA gene amplicon genera and fungal qPCR Ct values were utilized in a SPIEC-EASI co-occurrence network analysis as previously performed in RStudio using SpiecEasi version 1.2.4 (https://github.com/zdk123/SpiecEasi), devtools version 2.4.3 (https://CRAN.R-project.org/package=devtools), phyloseq version 1.4.0 (https://bioconductor.org/packages/phyloseq/), and igraph version 1.2.11 (https://CRAN.R-project.org/package=igraph)^18,38–40^. Specific SPIEC-EASI parameters included: Meinhausen–Bühlmann estimation method, lambda minimum ratio of 0.01, and nlambda of 20^38^. SPIEC-EASI utilized a neighborhood selection method termed Meinshausen and Bühlmann (MB)^38,41^. The MB method has been demonstrated to control FDR^42^. Correlations were performed for each stage (preweaning, nursery, and growth adult) with corresponding and fungal qPCR Ct values, according to individual samples (i.e. individual swine and single time point). Correlation plots were simplified to only correlations connected to the fungal node in each stage.

All bioinformatic scripts can be found in Supplemental Document S2.

### Ethics approval and consent to participate

Pigs were managed according to the Kansas State University Institutional Animal Care and Use Committee (IACUC) approved protocol #4036.

## Results and discussion

We collected a total of 234 samples across 31 time points (5 preweaning, 15 nursery, and 11 growth adult) from ten pigs (Supplemental Table S1). A total of 10,187,636 sequences resulted from sequencing; we recovered an average of 33,394 ASVs per sample following QIIME2 quality control. Out of the recovered ASVs, an average of 80.1% (79.7% bacteria and 0.4% archaea) populations were annotated with SILVA version 132 (Supplemental Table S4).

### Volatility in the preweaning stage preceded microbial establishment and stability in later growth stages

**Figure 1:**
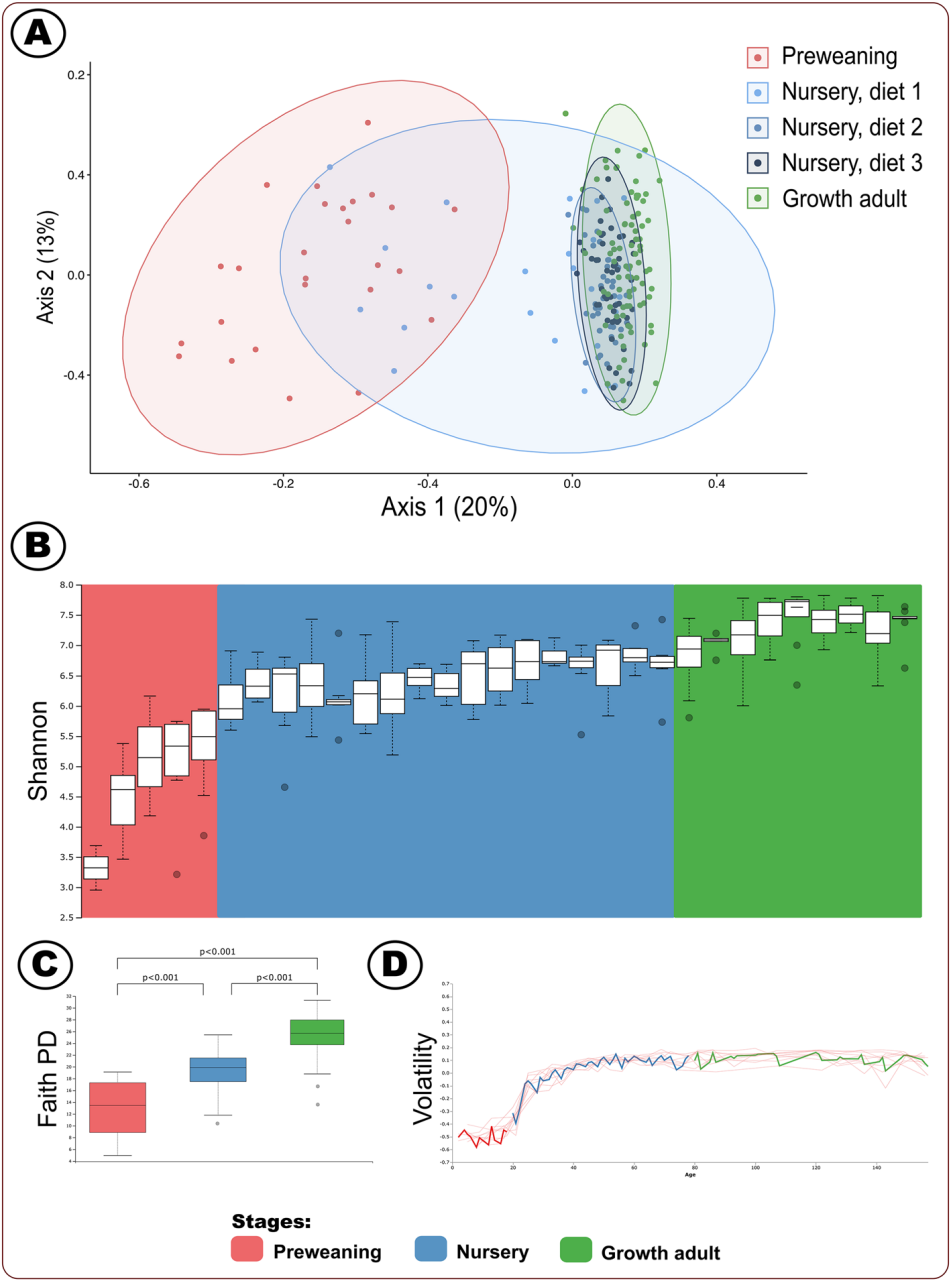
(**A**) Weighted uniFrac PCoA plot^29–32^ depicting composition; dots represent distinct samples. Nursery stage is separated according to the three diets fed during the stage. (**B**) Longitudinal Shannon diversity with Kruskal–Wallis statistical analysis^30^. (**C**) Faith’s phylogenetic diversity^30^ (PD). (**D**) Volatility control chart of the first axis of the PCoA^30^. Figure was edited in Inkscape version 1.0.2 (https://inkscape.org/)^88^.

As shown in Fig. 1A, the weighted UniFrac PCoA illustrated a distinct clustering of bacterial community composition between the three growth stages as the pig transitioned from young host to adult (Supplemental QIIME2 File S1: QIIME2 weighted unifrac PCoA, which can be uploaded and viewed at https://view.qiime2.org/). We further observed convergence amongst dietary clusters of the swine hosts in the nursery stage, with the two latter diet-stages of nursery being more similar to the growth adult hosts. We showed in our study that a young host lacked an established, shared microbiome, but converged with age and environmental changes such as diet and shared housing. The preweaning and first part of the nursery stages had the most divergent microbial composition amongst the individual swine. After this first nursery stage, the composition was relatively similar amongst the latter two nursery and growth stage swine. These patterns suggest the microbiome could be highly influenced by their respective diets during the different stages, and was rather stable once the microbial members had established^6–8^. Previous research has illustrated distinct microbial populations in swine hosts according to stage of development^6–8^.

Our ⍺ diversity results (Shannon index, Faith’s phylogenetic diversity, effective number of species (ENS), Shannon effective number, and Bray Curtis dissimilarity index) paralleled the PCoA analysis, indicating an establishing microbiome in the young swine (Fig. 1B,C, Supplemental Table S4, Supplemental QIIME2 File S2: Shannon diversity index, Supplemental QIIME2 File S3: ENS, and Supplemental QIIME2 File S4: Faith’s phylogenetic diversity; each QIIME2 file can be uploaded and viewed at https://view.qiime2.org/). We found that ⍺ diversity increased throughout the lifetime. We demonstrated the increasing diversity as overall stage comparisons (preweaning [P] vs nursery [N]: N vs growth adult [G]: and P vs G) were significantly different (p ≤ 0.001) for all ⍺ diversity measures according to PERMANOVA and Krustal-Wallis analyses. Studies have indicated this increase in microbial diversity during host development is typical across many different host species^1,2^. When we investigated the data longitudinally according to sampling day, the preweaning host demonstrated comparatively lower diversity which increased until weaning. This developmental diversity increase was followed with a relatively stable period during the nursery stage. We found distinctions in ⍺ diversity methods in the growth adult host. Shannon index and Faith’s phylogenetic diversity demonstrated small increases in the growth adult ⍺ diversity, whereas ENS and Shannon effective number illustrated a greater range of ⍺ diversity in the older swine. These distinctions in ⍺ diversity in the older swine should be further evaluated for an enhanced understanding of diversity in older swine, and how a wider range of diversity across swine could impact swine health.

Volatility results corroborated previous findings of a changing neonate microbiome which established in the weaned host (Supplemental QIIME2 File S5: volatility, which can be uploaded and viewed at https://view.qiime2.org/). Our microbial composition volatility index in the preweaning host hovered near −0.5 while approaching 0 in the early nursery stage (Fig. 1D). These volatility findings further suggested that the young preweaning host had a relatively more volatile, fluctuating microbiome. Our results were consistent with another mammalian study which demonstrated a volatile youth microbiome establishment period in children aged from birth to approximately 3 years of age^43^.

Together, our PCoA, diversity indices and volatility analyses suggested that the preweaning neonate host contained a developing gut microbiome which started establishing in the nursery stage. We showed that the microbiome was converging in the early nursery host, and there were comparatively fewer changes in microbial diversity after the convergence of the microbial community in the nursery host. We suggest that the forming and establishment of microbial populations during the preweaning and early nursery stages was likely crucial to the well-being of the swine host. Previous research demonstrates the importance of early microbiome dynamics as abnormal neonate gut microbiome development can result in diabetes, IBD and obesity^4,44^.

### Microbial-host stage development suggested metabolic and pathogenic potential associations

**Figure 2:**
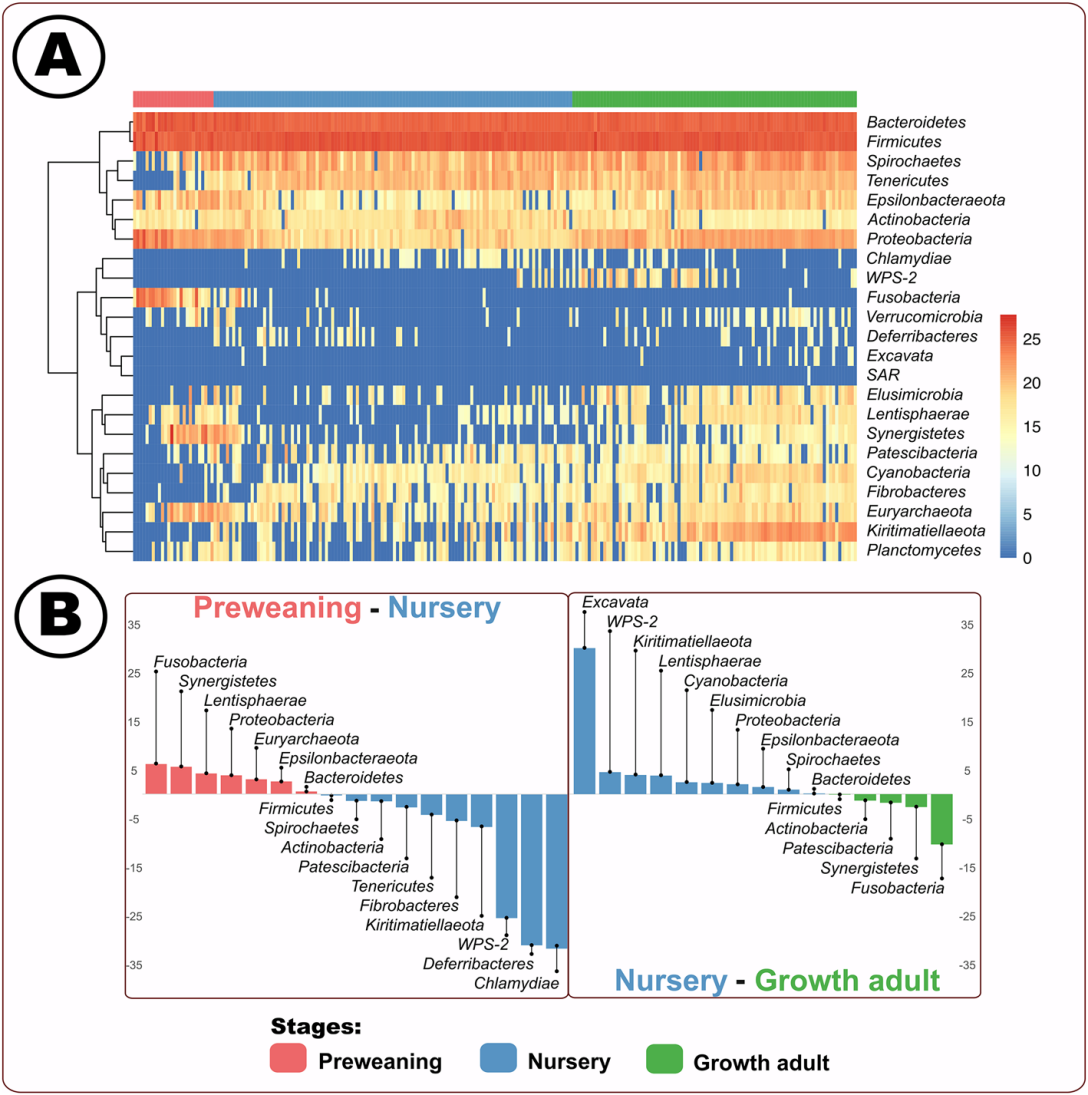
(**A**) Longitudinal heat map of DESeq2 resulting phyla relative abundances; each column represents a distinct sample^33,35,36^. (**B**) DESeq2 differentially identified (p < 0.05) phyla^33,35^. Figure was edited in Inkscape version 1.0.2 (https://inkscape.org/)^88^.

Our study supported previous bacterial establishment dynamics while elucidating novel stage-associations, highlighting a need for functional determination of the enteric microbiome according to host development. We analyzed the host microbial membership and identified 23 phyla (Fig. 2, Supplemental Table S5). We demonstrated a core bacterial population consisting of two phyla (*Bacteroidetes* and *Firmicutes*) which dominated throughout the lifetime of the swine host, suggesting these bacterial populations have essential implications to the host’s health and well-being^7,45,46^. Our study showed that *Bacteroidetes* and *Firmicutes* were the predominating core microbes (Fig. 2A). These results were consistent with findings from previous research demonstrating consistent domination of *Firmicutes* and *Bacteroidetes*^7,45,46^. *Firmicutes* and *Bacteroidetes* are known to metabolize carbohydrates into short chain fatty acids (SCFAs)^47,48^, suggesting that the two core phyla in our results have a wide range of beneficial attributes for the swine including acting as a cellular energy source, protecting DNA, and modulating diseases^49–51^. Therefore, given the necessity for energy and continual carbohydrate availability throughout the host lifetime, it is reasonable to identify *Firmicutes* and *Bacteroidetes* throughout the host life.

DESeq2 differential phyla distinctions between stages (adjusted p ≤ 0.05) suggested microbes were changing between preweaning and nursery stages but were relatively stable between nursery and growth adult swine. Two phyla identified in preweaning swine, compared to nursery swine, contained distinct microbes with potentially different metabolic implications: *Euryarchaeota* (log2-fold change: 3.06) and *Lentisphaerae* (log2-fold change: 4.26) (Fig. 2B). *Euryarchaeota* is an archaeon which has been associated with improved fiber digestion^52,53^. We hypothesized that microbes within the *Euryarchaeota* phylum were working alongside and with the bacterial community to shape the host microbiome, which can influence overall host health and well-being^54,55^. Alternatively, *Lentisphaerae* is thought to have a role in SCFA production resulting in a crucial source of energy for the swine host^56,57^. This differential identification of two carbohydrate metabolizing phyla, *Euryarchaeota* and *Lentisphaerae*, supports the different dietary sources of carbohydrates during the preweaning and nursery stages. Compared to the preweaning host, we identified three metabolic-associated phyla in the nursery host: *Deferribacteres* (log2-fold change: −31.09), *Fibrobacteres* (log2-fold change: −5.50), and *Tenericutes* (log2-fold change: −4.28). *Deferribacteres* is associated with diets containing iron^58–60^; *Fibrobacteres* is known for metabolizing non-soluble polysaccharides or carbohydrates^61^; and the function of *Tenericutes* remains elusive although the bacteria has been positively correlated with diets high in protein^62^. These three phyla remained unchanged between the nursery and growth adult swine suggesting that the microbes might perform similar metabolic roles during both developmental stages. Our observations of distinct microbial populations through the different stages of the pig paralleled the PCoA, diversity and volatility results, indicating a distinct gut microbiome composition and development during preweaning and early nursery. Considering previous research, we surmised that alongside bacteria and archaea establishment, microbial metabolic roles contributed to this stage-associated development under the influence of host factors, especially diet.

In addition, we also observed that the differential phyla indicated development of stage-dependent potential opportunistic pathogens (Fig. 2B). Preweaning-associated potential opportunistic pathogens included (Fig. 2B): *Fusobacteria* (log2-fold change: 6.2)^63–65^, *Synergistetes* (log2-fold change: 5.6)^66,67^, and *Proteobacteria* (log2-fold change: 3.8)^68,69^; nursery: *WPS-2* ([P vs N log2-fold change: −25.5][N vs G log2-fold change: 4.5])^70^, and *Spirochaetes* ([P vs N log2-fold change: −1.4][N vs G log2-fold change: 0.9])^71^; and growth adult: *Fusobacteria* (log2-fold change: −10.4)^63–65^ and *Synergistetes* (log2-fold change: −2.7)^66,67^. Interestingly, *Fusobacteria* and *Synergistetes* were found in both the preweaning and growth host. Further investigation is needed to evaluate the pathogenicity and determine the developmental significance of these phyla in the nursery growth swine.

**Figure 3:**
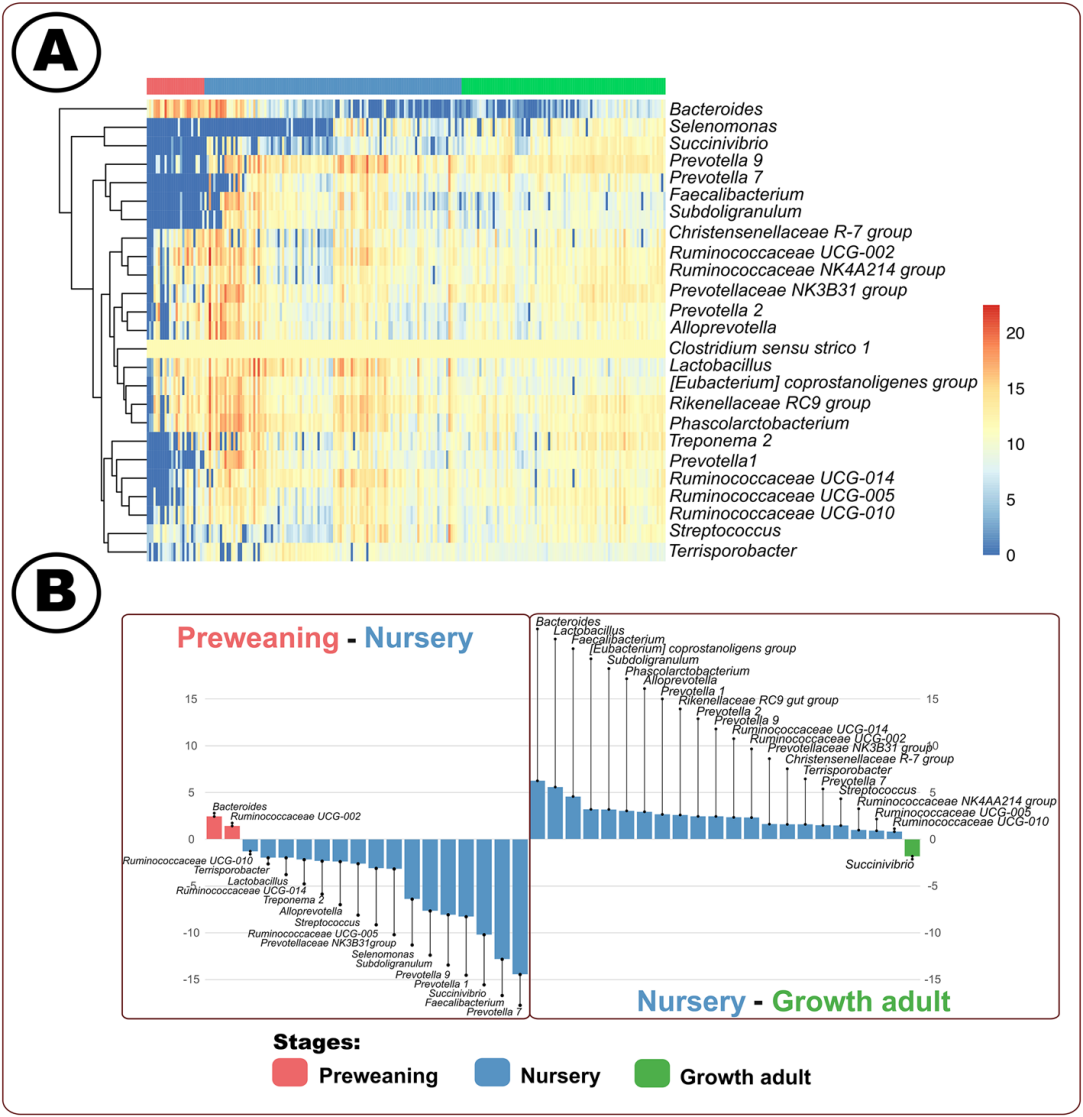
(**A**) Longitudinal heat map of DESeq2 resulting genera relative abundances; each column represents a distinct sample^33,35,36^. (**B**) DESeq2 differentially identified (p < 0.05) genera^33,35^. Figure was edited in Inkscape version 1.0.2 (https://inkscape.org/)^88^.

We identified 25 genera with an average relative abundance greater than 1% amongst the three stages (Fig. 3 and Supplemental Table S5). Unlike the phyla level analysis, we did not observe a core genus but instead identified three distinct clusters, based on tree branches and detection patterns, throughout the lifetime of the host (Fig. 3A). The first cluster consisted solely of *Bacteroides* as the bacterial population decreased post-weaning. *Succinivibrio* and *Selenomonas* appeared sporadically in the mid-nursery host followed by plateau in the growth adult stage. The final cluster, with 22 genera, generally appeared at a higher relative abundance earlier, than *Succinivibrio* and *Selenomonas*, in the preweaning or newly weaned host. Interestingly, although *Bacteroides*, *Succinivibrio*, and *Selenomonas* are all heavily reliant on carbohydrate utilization^72–74^, our data suggested that these genera were absent during different developmental stages. We hypothesized this could be related to these bacteria utilizing distinct carbohydrate sources^75–77^. Future research is necessary to evaluate how these bacterial species were utilizing dietary carbohydrates and interacting among the microbes and host.

The majority of stage-associated genera were identified in the nursery host which could indicate a need for specialized microbial roles in SCFA productions during this stage. *Bacteroides* ([P vs N log2-fold change: 2.4] [N vs G log2-fold change: 6.2]) was decreasing in relative abundance throughout the pig’s life stages (Fig. 3A,B)^72^. Conversely, *Succinivibrio* ([P vs N log2-fold change: −10.2;][N vs G log2-fold change: −1.8) was increasing through the stages^77^. The remaining potential SCFA-associated genera that were associated with the nursery host were *Faecalibacterium* ([P vs N log2-fold change: −12.8][N vs G log2-fold change: 4.5])^78^, *Prevotella 7* ([P vs N log2-fold change: −14.4][N vs G log2-fold change: 1.5])^79^, *Prevotella 1* ([P vs N log2-fold change: −8.2][N vs G log2-fold change: 2.6])^79^, *Subdoligranulum* ([P vs N log2-fold change: −7.6][N vs G log2-fold change: 3.1])^19^, *Prevotella 9* ([P vs N log2-fold change: −8.0][N vs G log2-fold change: 2.4])^80^, *Alloprevotella* ([P vs N log2-fold change: −2.4][N vs G log2-fold change: 2.9])^81^, *Prevotellaceae NK3B31 group* ([P vs N log2-fold change: −3.2][N vs G log2-fold change: 1.6])^82^, *Ruminococcaceae UCG-014* ([P vs N log2-fold change: −2.2][N vs G log2-fold change: 2.3])^83^, *Ruminococcaceae UCG-005* ([P vs N log2-fold change: −3.1][N vs G log2-fold change: 0.9])^83^, and *Ruminococcaceae UCG-010* ([P vs N log2-fold change: −1.3][N vs G log2-fold change: 0.8])^83^. The nursery host contained the most genera with SCFA metabolizing potential, suggesting that this is related to the microbiome dynamics as the microbes were working towards establishing. The bacteria populations within these genera associated with the nursery host could have taken advantage of the perturbations during these stages to proliferate. Akin to the SCFA potential metabolism findings, potential opportunistic pathogen genera were also only identified in nursery swine: *Streptococcus* ([P vs N log2-fold change: -2.6][N vs G log2-fold change: 1.4])^84^ and *Terrisporobacter* ([P vs N log2-fold change: -2.0][N vs G log2-fold change: 1.6])^85^. We observed that potential opportunistic pathogens were solely in the nursery host, suggesting that a turbulent microbiome enhanced the risk of pathogen development^10^. Although our present study provided insights into the microbial shifts during the different life stages of the swine, clearly there are complexities during microbiome establishment which warrant increased investigation. Further studies should elucidate how microbial metabolic roles and interactions influence microbiome establishment and pathogen prevalence.

### Temporal dynamics of Kazachstania slooffiae and association with bacterial diversity

**Figure 4:**
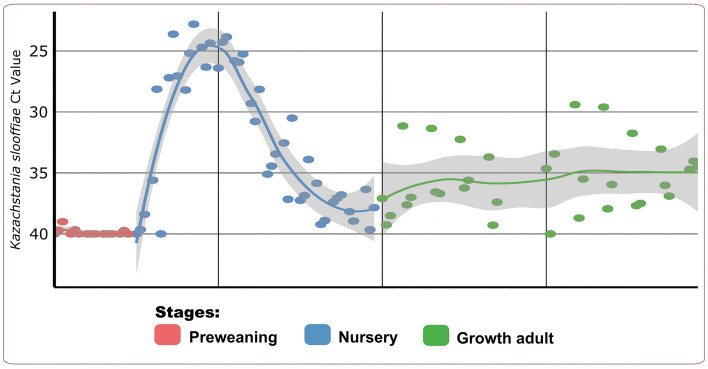
*Kazachstania slooffiae* qPCR Ct value according to day of age with line of best fit and 95% confidence interval by stage. Figure was edited in Inkscape version 1.0.2 (https://inkscape.org/)^88^.

Our findings suggested that fungal-bacterial interactions in the swine host could influence both bacteriome and mycobiome establishment and dynamics, therefore leading to the decline in *K. slooffiae* abundance in hosts. We performed qPCR and demonstrated varied *K. slooffiae* abundance according to developmental stage (Fig. 4). We noticed the fungus was absent in the preweaning host but its presence peaked in the nursery host from 25 to 46 days of age, with a steady decrease in abundance past 46 days of age. We determined fungal presence was more dispersed in the older host, as indicated by a larger 95% confidence interval. Interestingly, we found the increase in *K. slooffiae* coincided with the establishment of the microbiome near weaning. Previous studies have indicated an increase of *K. slooffiae* in the early nursery stage (swine hosts aged 21–35 days)^14,18^. *Kazachstania slooffiae* abundance past 35 days of age were previously unknown. Our findings showed that *K. slooffiae* abundance declined during the late nursery stage and plateaued in the growth adult stage, adding to the growing knowledge in the understanding of this fungi. Our fungal research suggested that *K. slooffiae* underwent stage-specific growth patterns, similar to that of the bacteriome. The factors which directly influenced *K. slooffiae* increase and decline are not yet known. Prior publications indicate associations between members within the microbiome, including between fungi and bacteria, may have implications to the well-being of the hosts^11^.

**Figure 5:**
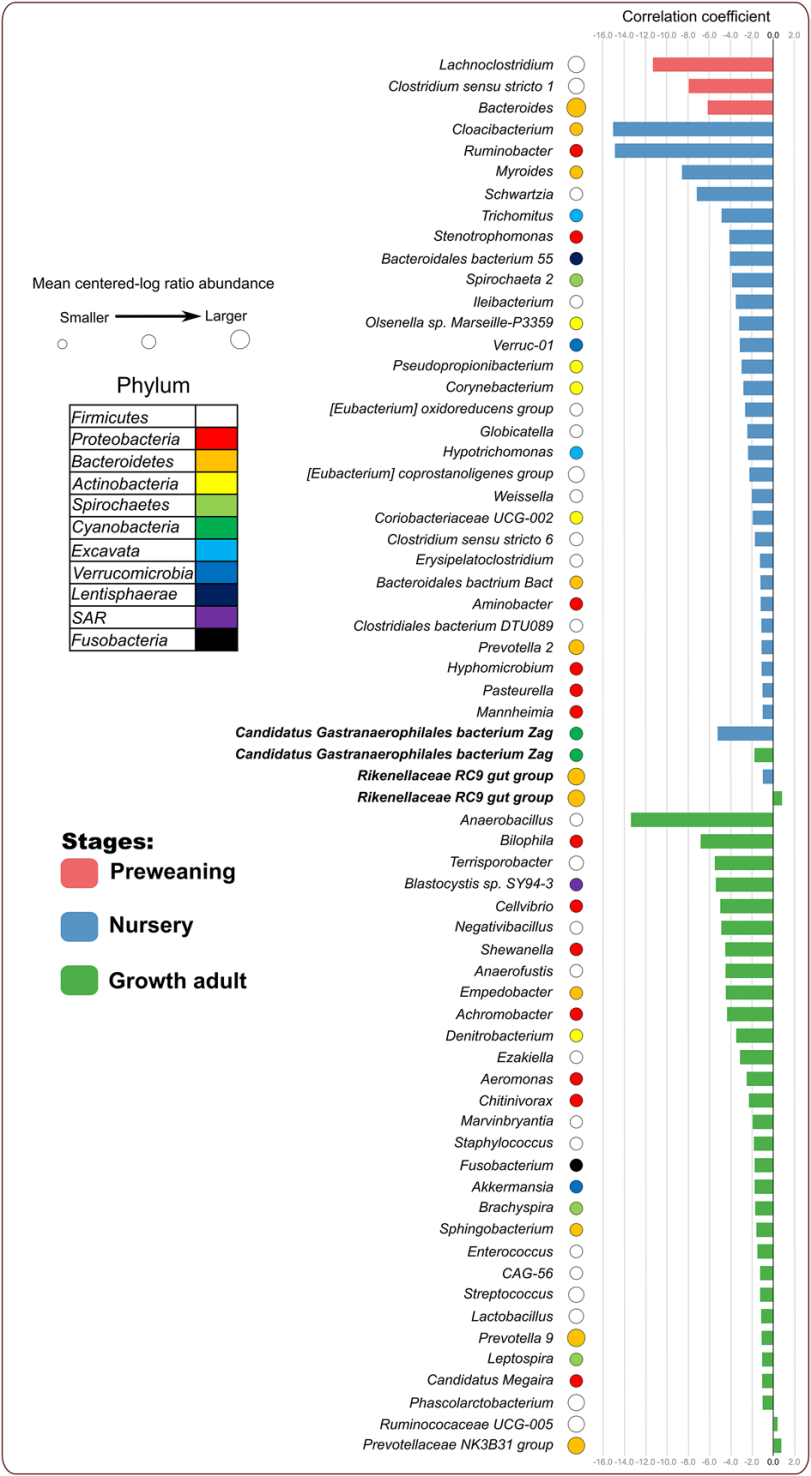
SPIEC-EASI correlation results between *Kazachstania slooffiae* and genera^18,38–40^. Figure was edited in Inkscape version 1.0.2 (https://inkscape.org/)^88^.

We performed taxonomic correlation analyses to further investigate fungi-bacteria interactions in the gut microbiome. Our increasing correlation network complexity with host age and lack of shared *K. slooffiae*-correlating genera across stages highlighted stage-dependent microbiome development. We simplified our correlation models to depict direct correlations between *K. slooffiae* and genera according to developmental stage (Fig. 5 and Supplemental Table S6). We identified 65 correlations (3 in preweaning, 30 nursery, and 32 growth adult). Previous research has indicated increasingly complex fungal-bacteria network correlations as both the microbiome and host develop from preweaning to nursery, but growth adult stage correlates were previously unknown^18^. We identified only two shared correlates between the nursery and growth adult stages: *Rikenellaceae RC9 gut group* and *Candidatus Gastranaerophilales bacterium Zag*. The significance of these genera, especially pertaining to *K. slooffiae*, are not understood and are a topic for future research. The lack of shared *K. slooffiae* correlating taxa may be related to stage-specific bacteria and stage-specific bacteriome–mycobiome interactions.

Our specific network correlation highlighted novel associations between *K. slooffiae* and the bacteriome throughout the host lifetime, suggesting the changes associated with weaning, including dietary change and stress, might have allowed for *K. slooffiae* expansion while the fungal decline may be attributed to competition with bacteria. Previous publications have identified eight correlations with *K. slooffiae*^18,19^. Our results included three out of the eight prior *K. slooffiae* correlations: *Lactobacillus* (correlation coefficient −1.2, growth adult), *Prevotella 9* (−1.2, growth adult), and *Prevotella 2* (−1.1, nursery)^19^. Previous research indicated positive correlations of *K. slooffiae* and *Lactobacillus*, *Prevotella 9*, and *Prevotella 2*, whereas our correlations were negative^19^. We surmised that negative correlation between *Lactobacillus* and *K. slooffiae* would be analogous to the inhibition of *Lactobacillus* growth by *Candida* in humans^11^. Previous research has identified genetic similarity between *K. slooffiae* and *Candida*^12,86^. Previous studies suggested that *Lactobacillus* may work alongside other bacteria to deter *Candida* growth, such as through short chain fatty acid production^11^. In fact, for our findings, the majority of our network correlations between *K. slooffiae* and genera were negative, with only three positive correlations (*Rikenellaceae RC9 gut group* (0.9), *Prevotellaceae NK3B31 group* (0.7), and *Ruminococcaceae UCG-005* (0.4)) were identified in growth hosts. Inverse abundances between fungi and bacteria are indicative of competition or amensalism^87^, which could explain the sharp decline of *K. slooffiae* populations in the nursery host (Fig. 4). We further hypothesized that the post-weaning increase of *K. slooffiae* might be attributed to the dietary change as *K. slooffiae* is unable to utilize milk galactose^12^. The dietary transition and host stress from preweaning to nursery might have allowed the increase in *K. slooffiae* populations, even with bacterial establishment relatively progressed^12,19^. Our correlation network results showed numerous (63 novel correlations, Fig. 5) novel *K. slooffiae* correlations which could aid in divulging establishment dynamics within the bacteriome and mycobiome.

## Conclusions

We provided a comprehensive evaluation of how bacteria and the fungus, *Kazachstania slooffiae,* developed through the different life stages of swine. The young preweaning host demonstrated comparatively low microbial diversity which increased near weaning. The growth adult host had a relatively similar microbiome overall compared to the nursery host, yet stage-specific associations, such as potential pathogens and fungal development, were noticed. We noticed the developing microbiome across hosts, even with differences in dam diet and parity status. Future research, with more swine, are crucial to determining the extent to which these dam factors and stage-associated characteristics influence microbiome development dynamics. Our findings provided a foundation for gut microbiome studies.

While microbial inter-kingdom interactions are known to have implications on host health, the intricacies of dynamics between bacteria and fungi are not well understood. We determined that distinct microbial taxa, diversity, and bacterial-fungi correlations were associated with different stages of life. These stage-associated attributes indicated there could be further stage-associated characteristics such as illness-inducing pathogens and energy providing carbohydrate metabolizing microbes. Future research is crucial to understand the interplay amongst microbes, especially on the functional level pertaining to carbohydrate utilization and relating these findings back to host health. As we evaluated general-swine host stage, additional research is also necessary to attribute specific host growth, development, and environmental factors, such as diet and housing, to the diversity changes we identified.

## Supplementary Information

Supplementary Information 1.

Supplementary Information 2.

Supplementary Information 3.

Supplementary Information 4.

Supplementary Information 5.

Supplementary Information 6.

Supplementary Information 7.

Supplementary Information 8.

Supplementary Information 9.

Supplementary Information 10.

Supplementary Information 11.

Supplementary Information 12.

Supplementary Table S2.

Supplementary Table S2.

Supplementary Table S3.

Supplementary Table S4.

Supplementary Table S5.

Supplementary Table S6.

Supplementary Table S7.

## Data Availability

The dataset(s) supporting the conclusions of this article is(are) available in the NCBI repository, BioProject PRJNA798835, https://www.ncbi.nlm.nih.gov/bioproject/?term=PRJNA798835.
