# Supplementary material for: Stability and volatility shape the gut bacteriome and Kazachstania slooffiae dynamics in preweaning, nursery and adult pigs
Source: Sci Rep. 2022 Sep 5;12:15080. doi: 10.1038/s41598-022-19093-9 (PMC9445069; doi:10.1038/s41598-022-19093-9)
Supplement: Supplementary file 1 — Supplementary Information 1. [file 41598_2022_19093_MOESM1_ESM.pdf]

Supplemental Document S1

General Observations Scoring

Table of Contents

Description 1
Chart 2

Swine General Observations Scoring System Description
Fecal Collection for Temporal Microbiome Assay

Systemic Behaviors

Apathy-lack of interest, enthusiasm, or concern
Depression/Anorexia-little interest in what is happening around them/lack of appetite: off feed (not eating)

General Appearance

Gaunt-overall appearance and body condition are abnormal and sickly looking. Skin may be different color, ears dropping, body may looking hairy instead of smooth

Respiratory

Tachypnea-abnormally rapid breathing; look for rise and fall of chest
Severe dyspnea-very difficult or labored breathing

Diarrhea

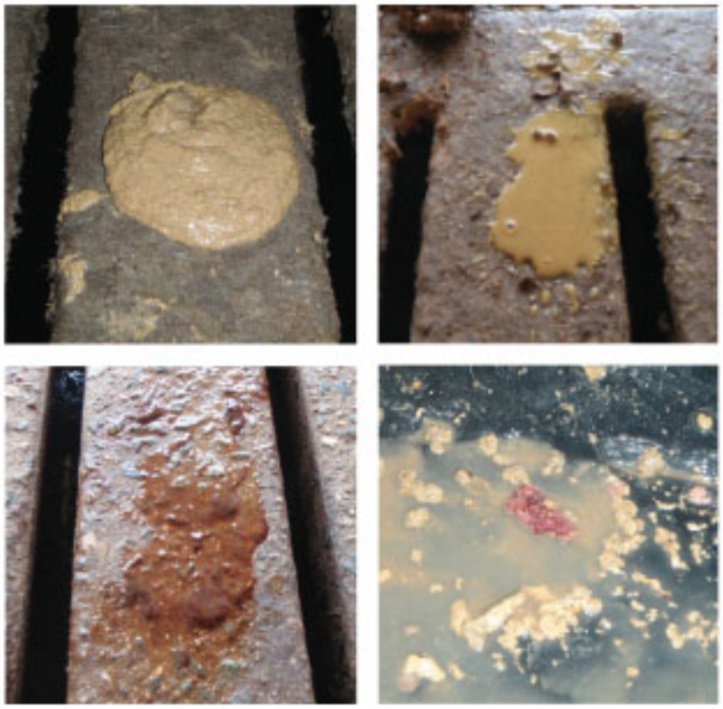

Pasty
semi-solid; wet-cement consistency

Runny/ Watery

Bloody

Mucoid
Larger amounts of mucus in stool

General Observations Scoring System Chart

Fecal Collection for Temporal Microbiome Assay

Observer(s): ____________________________ Date: __________________

**Parameters**	**Appearance**	**Score**	**Pig Number**									
			**1**	**2**	**3**	**4**	**5**	**6**	**7**	**8**	**9**	**10**
Systemic behaviors	Normal	0										
	Apathy	1										
	Depression/Anorexia	2										
	Severe depression	3										
General appearance	Normal	0										
	Gaunt	1										
Respiratory	Normal	0										
	Sneezing/Coughing	1										
	Tachypnea	2										
	Severe dyspnea	3										
Diarrhea	Normal (no diarrhea present)	0										
	Pasty (semi-solid; wet-cement consistency)	1										
	Runny/Watery	2										
	Mucoid	3										
	Bloody	4										
		**Weight (lbs)**										
		**Temperature (oF)**										
		**Flank-to-Flank (cm)**										

Comments/Procedures:
