# Supplementary material for: Stability and volatility shape the gut bacteriome and Kazachstania slooffiae dynamics in preweaning, nursery and adult pigs
Source: Sci Rep. 2022 Sep 5;12:15080. doi: 10.1038/s41598-022-19093-9 (PMC9445069; doi:10.1038/s41598-022-19093-9)
Supplement: Supplementary file 2 — Supplementary Information 2. [file 41598_2022_19093_MOESM2_ESM.pdf]

Supplemental Document S2

Bioinformatic Scripts

Table of Contents

QIIME2 1
Principal Coordinate Analysis (PCoA) 7
Faith and Effective Number of Species (ENS) Diversity Results 9
Shannon Transformation to Effective Number 10
Heatmaps and DESeq2 11
- Phyla 11
- Genera 12

SPIEC-EASI 15
- Preweaning 16
- Nursery 18
- Growth Adult 20

Associated Files

Supplemental Table S2

Kazachstania slooffiae qPCR results, components, and conditions[10].

Supplementary Table S7

Files for bioinformatic scripts.

Supplemental QIIME2 File S6

QIIME2 weighted unifrax PCoA results.

Supplemental QIIME2 File S7

Faith's phylogenetic diversity results.

Supplemental QIIME2 File S8

Effective number of species (ENS) results.

Supplemental QIIME2 File S9

Shannon diversity index results.

Supplemental QIIME2 File S10

QIIME2 Silva 132 taxonomic results.

<u>QIIME2</u>
Associated files: Supplementary Table S7 tabs: “Metadata_QIIME2_wNC”, “Metadata_QIIME2”, and “Metadata_QIIME2_NC”

```
#!/bin/bash -l
#SBATCH --job-name=swine_downstream

#SBATCH --mem-per-cpu=4G
#SBATCH --time=1-00:00:00
#SBATCH --ntasks=10
#SBATCH --nodes=1

#SBATCH --mail-user=qinghong@ksu.edu
#SBATCH --mail-type=ALL

module load QIIME2/2019.7
##Quality control on run20201112
cd /bulk/leet1/Swine_microbiome/KSU/Marker_genes/01_16S_Analysis/QC_run20201112
qiime tools import \
  --type 'SampleData[PairedEndSequencesWithQuality]' \
  --input-path
/bulk/leet1/Swine_microbiome/KSU/Marker_genes/00_RAWDATA/16S/run20201112 \
  --input-format CasavaOneEightSingleLanePerSampleDirFmt \
  --output-path demux-paired-end_run1.qza
qiime demux summarize \
  --i-data demux-paired-end_run1.qza \
  --o-visualization demux-paired-end_run1.qzv
qiime cutadapt trim-paired \
  --i-demultiplexed-sequences demux-paired-end_run1.qza \
  --p-front-f ^GTGCCAGCMGCCGCGGTAA \
  --p-front-r ^GGACTACHVGGGTWTCTAAT \
  --p-error-rate 0.1 \
  --p-discard-untrimmed True \
  --o-trimmed-sequences demux-primer-trimmed-end_run1.qza
qiime demux summarize \
  --i-data demux-primer-trimmed-end_run1.qza \
  --o-visualization demux-primer-trimmed-end_run1.qzv
qiime dada2 denoise-paired \
  --i-demultiplexed-seqs demux-primer-trimmed-end_run1.qza \
  --p-trunc-len-f 225 \
  --p-trunc-len-r 225 \
  --p-trim-left-f 0 \
  --p-trim-left-r 0 \
  --p-n-threads 10 \
  --o-table table_run1.qza \
  --o-representative-sequences rep-seqs_run1.qza \
```

```
  --o-denoising-stats denoising-stats_run1.qza
qiime metadata tabulate \
  --m-input-file denoising-stats_run1.qza \
  --o-visualization denoising-stats_run1.qzv


##Quality control on run20210216
cd /bulk/leet1/Swine_microbiome/KSU/Marker_genes/01_16S_Analysis/QC_run20210216
qiime tools import \
  --type 'SampleData[PairedEndSequencesWithQuality]' \
  --input-path
/bulk/leet1/Swine_microbiome/KSU/Marker_genes/01_16S_Analysis/QC_run20210216/pe-32-
manifest \
  --input-format PairedEndFastqManifestPhred33V2 \
  --output-path demux-paired-end_run2.qza
qiime demux summarize \
  --i-data demux-paired-end_run2.qza \
  --o-visualization demux-paired-end_run2.qzv
qiime cutadapt trim-paired \
  --i-demultiplexed-sequences demux-paired-end_run2.qza \
  --p-front-f ^GTGCCAGCMGCCGCGGTAA \
  --p-front-r ^GGACTACHVGGGTWTCTAAT \
  --p-error-rate 0.1 \
  --p-discard-untrimmed True \
  --o-trimmed-sequences demux-primer-trimmed-end_run2.qza
qiime demux summarize \
  --i-data demux-primer-trimmed-end_run2.qza \
  --o-visualization demux-primer-trimmed-end_run2.qzv
qiime dada2 denoise-paired \
  --i-demultiplexed-seqs demux-primer-trimmed-end_run2.qza \
  --p-trunc-len-f 225 \
  --p-trunc-len-r 225 \
  --p-trim-left-f 0 \
  --p-trim-left-r 0 \
  --p-n-threads 10 \
  --o-table table_run2.qza \
  --o-representative-sequences rep-seqs_run2.qza \
  --o-denoising-stats denoising-stats_run2.qza
qiime metadata tabulate \
  --m-input-file denoising-stats_run2.qza \
  --o-visualization denoising-stats_run2.qzv


###Quality control on run20210218
cd /bulk/leet1/Swine_microbiome/KSU/Marker_genes/01_16S_Analysis/QC_run20210218
qiime tools import \
```

```
  --type 'SampleData[PairedEndSequencesWithQuality]' \
  --input-path
/bulk/leet1/Swine_microbiome/KSU/Marker_genes/01_16S_Analysis/QC_run20210218/pe-32-
manifest \
  --input-format PairedEndFastqManifestPhred33V2 \
  --output-path demux-paired-end_run3.qza
qiime demux summarize \
  --i-data demux-paired-end_run3.qza \
  --o-visualization demux-paired-end_run3.qzv
qiime cutadapt trim-paired \
  --i-demultiplexed-sequences demux-paired-end_run3.qza \
  --p-front-f ^GTGCCAGCMGCCGCGGTAA \
  --p-front-r ^GGACTACHVGGGTWTCTAAT \
  --p-error-rate 0.1 \
  --p-discard-untrimmed True \
  --o-trimmed-sequences demux-primer-trimmed-end_run3.qza
qiime demux summarize \
  --i-data demux-primer-trimmed-end_run3.qza \
  --o-visualization demux-primer-trimmed-end_run3.qzv
qiime dada2 denoise-paired \
  --i-demultiplexed-seqs demux-primer-trimmed-end_run3.qza \
  --p-trunc-len-f 225 \
  --p-trunc-len-r 225 \
  --p-trim-left-f 0 \
  --p-trim-left-r 0 \
  --p-n-threads 10 \
  --o-table table_run3.qza \
  --o-representative-sequences rep-seqs_run3.qza \
  --o-denoising-stats denoising-stats_run3.qza
qiime metadata tabulate \
  --m-input-file denoising-stats_run3.qza \
  --o-visualization denoising-stats_run3.qzv


##Merge all data and carry out analysis
cd /bulk/leet1/Swine_microbiome/KSU/Marker_genes/01_16S_Analysis/Merge_Analysis
qiime feature-table merge \
  --i-tables
/bulk/leet1/Swine_microbiome/KSU/Marker_genes/01_16S_Analysis/QC_run20201112/table_r
un1.qza \
  --i-tables
/bulk/leet1/Swine_microbiome/KSU/Marker_genes/01_16S_Analysis/QC_run20210216/table_r
un2.qza \
  --i-tables
/bulk/leet1/Swine_microbiome/KSU/Marker_genes/01_16S_Analysis/QC_run20210218/table_r
un3.qza \
```

```
  --p-overlap-method 'sum' \
  --o-merged-table table.qza
qiime feature-table merge-seqs \
  --i-data
/bulk/leet1/Swine_microbiome/KSU/Marker_genes/01_16S_Analysis/QC_run20201112/rep-seqs_run1.qza \
  --i-data
/bulk/leet1/Swine_microbiome/KSU/Marker_genes/01_16S_Analysis/QC_run20210216/rep-seqs_run2.qza \
  --i-data
/bulk/leet1/Swine_microbiome/KSU/Marker_genes/01_16S_Analysis/QC_run20210218/rep-seqs_run3.qza \
  --o-merged-data rep-seqs.qza
qiime feature-table summarize \
  --i-table table.qza \
  --o-visualization table.qzv \
  --m-sample-metadata-file Metadata_QIIME2_wNC.txt
qiime feature-table tabulate-seqs \
  --i-data rep-seqs.qza \
  --o-visualization rep-seqs.qzv
qiime phylogeny align-to-tree-mafft-fasttree \
  --i-sequences rep-seqs.qza \
  --o-alignment aligned-rep-seqs.qza \
  --o-masked-alignment masked-aligned-rep-seqs.qza \
  --o-tree unrooted-tree.qza \
  --o-rooted-tree rooted-tree.qza
qiime diversity core-metrics-phylogenetic \
  --i-phylogeny rooted-tree.qza \
  --i-table table.qza \
  --p-sampling-depth 11105 \
  --m-metadata-file Metadata_QIIME2_wNC.txt \
  --output-dir core-metrics-results
qiime diversity alpha-group-significance \
  --i-alpha-diversity core-metrics-results/observed_otus_vector.qza \
  --m-metadata-file Metadata_QIIME2.txt \
  --o-visualization core-metrics-results/observed_otus_vector-group-significance.qzv
qiime diversity alpha-group-significance \
  --i-alpha-diversity core-metrics-results/faith_pd_vector.qza \
  --m-metadata-file Metadata_QIIME2.txt \
  --o-visualization core-metrics-results/faith-pd-group-significance.qzv
qiime diversity alpha-group-significance \
  --i-alpha-diversity core-metrics-results/shannon_vector.qza \
  --m-metadata-file Metadata_QIIME2.txt \
  --o-visualization core-metrics-results/shannon-group-significance.qzv
qiime diversity beta-group-significance \
  --i-distance-matrix core-metrics-results/bray_curtis_distance_matrix.qza \
```

```
  --m-metadata-file Metadata_QIIME2.txt \
  --m-metadata-column Stage \
  --o-visualization core-metrics-results/bray_curtis-Stage-significance.qzv
qiime diversity alpha-rarefaction \
  --i-table table.qza \
  --i-phylogeny rooted-tree.qza \
  --p-max-depth 11105 \
  --m-metadata-file Metadata_QIIME2_wNC.txt \
  --o-visualization core-metrics-results/alpha-rarefaction.qzv
qiime feature-classifier classify-sklearn \
  --i-classifier /bulk/qinghong/16S_Abby/silva-132-99-515-806-nb-classifier.qza \
  --i-reads rep-seqs.qza \
  --o-classification taxonomy_silva132.qza
qiime metadata tabulate \
  --m-input-file taxonomy_silva132.qza \
  --o-visualization taxonomy_silva132.qzv
qiime feature-table filter-samples \
  --i-table table.qza \
  --m-metadata-file Metadata_QIIME2_NC.txt \
  --p-exclude-ids True \
  --o-filtered-table table_NC_filtered.qza
qiime taxa barplot \
  --i-table table_NC_filtered.qza \
  --i-taxonomy taxonomy_silva132.qza \
  --m-metadata-file Metadata_QIIME2.txt \
  --o-visualization taxa-bar-plots_silva132.qzv

##Estimated number of species
cd
/bulk/leet1/Share_LeeLab/Swine_microbiome/KSU/Marker_genes/01_16S_Analysis/Merge_An
alysis
source /homes/qinghong/miniconda3/etc/profile.d/conda.sh
conda activate qiime2-2022.2
qiime feature-table filter-samples \
  --i-table table.qza \
  --m-metadata-file filtered_metadata_ENS.txt \
  --p-exclude-ids True \
  --o-filtered-table table_ENS_filtered.qza
qiime diversity alpha \
  --i-table table_ENS_filtered.qza \
  --p-metric enspie \
  --o-alpha-diversity ENS_vector.qza
qiime diversity alpha-group-significance \
  --i-alpha-diversity ENS_vector.qza \
  --m-metadata-file Swine_metadata_ENS.txt \
  --o-visualization ENS-group-significance.qzv
```

Principal Coordinate Analysis (PCoA)

```
# Associated files: Supplemental_QIIME2_File_S6.qza and Supplementary Table S7 tab
"Metadata_PCoA_and_Effective_Number"
# Open RStudio

# Install applications, if not installed previously
install.packages("tidyverse")
install.packages("qiime2R")
install.packages("plyr")
install.packages("ggpubr")

# Set working directory
setwd("QIIME2_Merge_Analysis/")

# Load applications
library("tidyverse")
library("qiime2R")
library ("plyr")
library("ggpubr")

#Generate PCoA plot points file
wunifrac<-read_qza("Supplemental_QIIME2_File_S6.qza")
SampleID <- ldply(wunifrac$data$Vectors$SampleID, data.frame)
PC1 <- ldply(wunifrac$data$Vectors$PC1, data.frame)
PC2 <- ldply(wunifrac$data$Vectors$PC2, data.frame)
merge <- c %>% add_column(PC1) %>% add_column(PC2)
write.csv(merge,"PCoA.csv")

# Combine the metadata ("Metadata_QIIME2" tab in Supplementary Table S7) and PCoA plot
points (PCoA.csv) into one file and save as Metadata_PCoA_Combined.csv (already performed
and generated tab "Metadata_PCoA_Combined.csv" in Supplemental Table S7)

Data <- read.csv("Swine_16S_Metadata-PCoA.csv",header=T)

#Generate PCoA plot
     ggscatter(Data, x = "PCoA_Axis_1", y = "PCoA_Axis_2",
          fill = "Stage_Diet",
    shape = 21,
          size = 2,
          ellipse = T,
          color = "Stage_Diet",
          palette = c(P = rgb(0.86, 0.44, 0.43),
                N.1 = rgb(0.49, 0.69, 0.92),
          N.2 = rgb(0.40, 0.57, 0.75),
          N.3 = rgb(0.20, 0.28,0.38),
          G = rgb(0.41, 0.68, 0.34)),
```

```
                xlab = "Axis 1 (20%)", ylab = "Axis 2 (13%)")
# Exported with width x height: 1000 x 651
# Close RStudio
```

Faith and Effective Number of Species (ENS) Diversity Results

```
# Associated files: Supplemental_QIIME2_File_S7.qza and Supplemental_QIIME2_File_S8.qza
# Open RStudio

# Install applications, if not installed previously
install.packages("qiime2R")

# Set working directory
setwd("QIIME2_Merge_Analysis/")

# Load applications
library("qiime2R")

# Generate alpha diversity result files
index_Faith<-read_qza("Supplemental_QIIME2_File_S7.qza")
index_Faith_values<-index_Faith$data
index_ENS<-read_qza("Supplemental_QIIME2_File_S8.qza")
index_ENS_values<-index_ENS$data

# Save files
write.csv(index_Faith_values,"Faith.csv")
write.csv(index_ENS_values,"ENS.csv")
```

Shannon Transformation to Effective Number

```
# Associated files: Supplemental_QIIME2_File_S9.qza and Supplementary Table S7 tab
“Metadata”
# Open RStudio

# Install applications, if not installed previously
install.packages("tidyverse")
install.packages("qiime2R")

# Set working directory
setwd("QIIME2_Merge_Analysis/")

# Load applications
library("tidyverse")
library("qiime2R")

# Generate Shannon diversity index plot points file
index<-read_qza("Supplemental_QIIME2_File_S6.qza")
index_values<-index$data

# Transform Shannon diversity index into effective number
index_values$shannon = exp(index_values$shannon)
write.csv(index_values,"Effective_Number.csv")

# Combine the metadata (“Metadata” tab in Supplementary Table S7) and effective number values
("Effective_Number.csv") into one file and save as Metadata-Effective_Number_Combined.csv
(already performed and generated tab “Metadata-Effective_Number_Combined.csv” in
Supplemental Table S7)

Data <- read.csv("Metadata-Effective_Number_Combined.csv",header=T)
Data$Day <- as.character(Data$Day)

# Generate box plot
Data %>%
mutate(name = fct_relevel(as.factor(Day), "30920", "31320", "31720", "32120", "32520",
"32920", "40220", "40620", "41020", "41420", "41820", "42220", "42620", "43020", "50420",
"50820", "51220", "51620", "81120", "52020", "52420", "52820", "60220", "60920","61620",
"62320", "70720", "71420", "72120", "72820", "80420"))

ggplot(Data, aes(group=Day, y=Effective_Number)) +
   geom_boxplot(fill="slateblue", alpha=0.2) +
   theme(axis.text.x=element_blank(), axis.ticks.x=element_blank()) +
   ylab("Effective Number")
# Export as svg image
# Close RStudio
```

Heatmaps and DESeq2
Associated files: Supplemental_QIIME2_File_S10.qzv and Supplementary Table S7 tabs: "Metadata", "leve_2", "level_6_RA>1%"

Phyla
Prepare Files for Heatmap and DESeq2
- Prepare count matrix
    - Use level 2 csv file from Supplemental_QIIME2_File_S7.qzv
        - Go to https://view.qiime2.org/ on your web browser
        - Upload taxa-bar-plots_silva152.qzv file
        - Navigate to level 2
        - 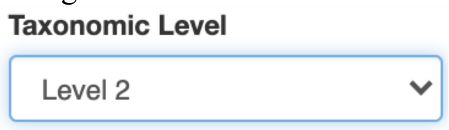

        - Download csv file
        - 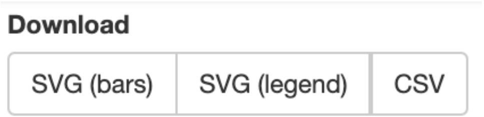

    - Open a new Excel file and the csv file (level-2.csv)
    - Copy the contents of the csv and paste into the Excel file
    - Copy the Excel file contents, make a new tab in Excel and paste transposed (right click > "Paste Special" > "Transpose")
        - Taxa should now be in rows with one sample per column
    - Delete the first Excel tab (non-transposed data)
    - Delete the rows with "D_0__Bacteria;__" and "Unassigned;__" (both are unclassified bacteria)
    - Normalize data
        - Sum each column (total reads per sample)
        - Divide each cell by the total number of reads per sample and multiple by a multiple of 10
            - If total reads were 999,888,777, then multiply by 1,000,000,000
            - If total reads were 999,888,777,666, then multiply by 1,000,000,000,000
        - Use round =ROUND(CELL_NUMBER,0) Excel function to generate an integer for each cell
        - Copy and paste the last block of cells (rounded numbers) as values
        - Delete all other rows except first row and rounded/integers rows
            - Can check math by summing each column which should be a multiple of 10
            - Do not save the file with this summation
    - Save the Excel file as a csv file (level_2.csv)
    - Close web browser and Excel

Prepare for DESeq2
Open R studio
Install applications, if not already installed

```
if (!requireNamespace("BiocManager", quietly = TRUE))
    install.packages("BiocManager")

BiocManager::install("DESeq2")

# Enter "a" when asked "Update all/some/none? [a/s/n]:"
# Enter "Yes" when asked "Do you want to install from sources the package which needs
compilation? (Yes/no/cancel)"

if (!requireNamespace("BiocManager", quietly = TRUE))
    install.packages("BiocManager")

BiocManager::install("pheatmap")

# Set working directory
setwd("DESeq2/")

# Load applications and files
library("DESeq2")
library("pheatmap")
coldata <- read.table("Metadata.csv", sep=",", header=TRUE)
cts_lvl2 <- read.table("level_2.csv", sep=",", header=TRUE, row.names=1)

# Perform differential analysis
dds_lvl2 <- DESeqDataSetFromMatrix(countData = cts_lvl2,
                                   colData = coldata,
                                   design= ~ Stage)
dds_lvl2 <- DESeq(dds_lvl2)

# Generate Heatmap
select_lvl2 <- order(rowMeans(counts(dds_lvl2,normalized=TRUE)),
                     decreasing=TRUE)[1:20]
df_lvl2 <- as.data.frame(colData(dds_lvl2)[,c("Stage")])
ntd_lvl2 <- normTransform(dds_lvl2)
rownames(df_lvl2) <- colnames(ntd_lvl2)
pheatmap(assay(ntd_lvl2), cluster_rows=TRUE, show_rownames=TRUE,
         cluster_cols=FALSE, annotation_col=df_lvl2)
pheatmap(assay(ntd_lvl2), cluster_rows=TRUE, show_rownames=T,
         cluster_cols=FALSE, annotation_col=df_lvl2, show_colnames = FALSE)
```

Genera

Prepare Files for Heatmap and DESeq2

- Prepare count matrix
  - Use level 6 csv file from Supplemental_QIIME2_File_S7.qzv
    - Go to https://view.qiime2.org/
    - Upload taxa-bar-plots_silva152.qzv file

        - Navigate to level 6 (figure shows 2, but navigate to 6)
        - 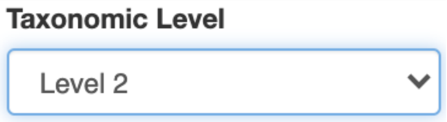

        - Download csv file
        - 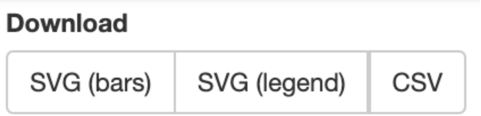

    - Open a new Excel file and the csv file (level-6.csv)
    - Copy the contents of the csv and paste into the Excel file
    - Copy the Excel file contents, make a new tab in Excel and paste transposed (right click > “Paste Special” > “Transpose”)
        - Taxa should not be in rows with one sample per column
    - Delete the first Excel tab (non-transposed data)
    - Delete the rows without a sixth level (only show D_4 or higher levels), rows with non-specific genera (i.e. D_5_uncultured bacterium), and rows with chloroplast, swine or mitochondria domains
    - Normalize data
        - Sum each column (total reads per sample)
        - Divide each cell by the total number of reads per sample and multiple by a multiple of 10
            - If total reads were 999,888,777, then multiply by 1,000,000,000
            - If total reads were 999,888,777,666, then multiply by 1,000,000,000,000
        - Use round =ROUND(CELL_NUMBER,0) Excel function to generate an integer for each cell
        - Copy and paste the last block of cells (rounded numbers) as values
        - Delete all other rows except first row and rounded/integers rows
            - Can check math by summing each column which should be a multiple of 10
            - Do not save the file with this summation
    - Combine all rows with less than 1% abundance into “other” category
    - Save the Excel file as a csv file (level-6.csv)

```
# Load file
cts_lvl6 <- read.table("level_6_RA>1%.csv", sep=",", header=TRUE, row.names=1)

# Perform differential analysis
dds_lvl6 <- DESeqDataSetFromMatrix(countData = cts_lvl6,
                     colData = coldata,
                     design= ~ Stage)
dds_lvl6 <- DESeq(dds_lvl6)

# Generate Heatmap
select_lvl6 <- order(rowMeans(counts(dds_lvl6,normalized=TRUE)),
          decreasing=TRUE)[1:20]
```

```
df_lvl6 <- as.data.frame(colData(dds_lvl6)[,c("Stage")])
ntd_lvl6 <- normTransform(dds_lvl6)
rownames(df_lvl6) <- colnames(ntd_lvl6)
pheatmap(assay(ntd_lvl6), cluster_rows=TRUE, show_rownames=TRUE,
        cluster_cols=FALSE, annotation_col=df_lvl6)
pheatmap(assay(ntd_lvl6), cluster_rows=TRUE, show_rownames=T,
        cluster_cols=FALSE, annotation_col=df_lvl6, show_colnames = FALSE)

# Close RStudio
```

SPIEC-EASI

Associated files: Supplemental Table S2 “Results” tab, Supplementary Table S7 tabs: “level_6_all_RA”, “Metadata”, “level_6_with_KS_PCR_P”, “level_6_Taxonomy_P”, “net1df_6_P”, “net1df_KS_6_P”, “vsize_6_P”, “net1df_KS_6_P”, “level_6_with_KS_PCR_N”, “level_6_Taxonomy_N”, “net1df_6_N”, “net1df_KS_6_N”, “vsize_6_N”, “net1df_KS_6_N”, “level_6_with_KS_PCR_G”, “level_6_Taxonomy_G”, “net1df_6_G”, “net1df_KS_6_G”, “vsize_6_G”, and “net1df_KS_6_G”

Prepare taxonomic files according to stage

- Utilize “level_6_all_RA” and “Metadata” tabs in in Supplementary Table S7 to generate files which contain read counts and qPCR values (Supplemental_Table_S2; generate “level_6_with_KS_PCR” files) for taxa identified in each stage (P, N, G) (generate “Taxonomy” files)
- Include *Kazachstania slooffiae*
- Above steps already performed, and final files (which should be saved as csv files) are tabs in Supplementary Table S7 : “level_6_with_KS_PCR_P”, “level_6_Taxonomy_P”, “level_6_with_KS_PCR_N”, “level_6_Taxonomy_N”, “level_6_with_KS_PCR_G”, and “level_6_Taxonomy_G”

```
# Open RStudio

# Install applications, if not installed previously
install.packages("devtools")
install.packages("SpiecEasi")
install.packages("phyloseq")
install.packages("igraph")
```

```
# Set working directory
    setwd("SPIEC-EASI/")

# Load applications and file
library("devtools")
library("SpiecEasi")
library("phyloseq")
library("igraph")
sampledata <- read.csv("Metadata.csv", row.names=1)
```

Preweaning

```
# Load additional files for preweaning
otumat.6.P <-(read.csv("level_6_with_KS_PCR_P.csv", row.names=1))
taxmat.6.P <- as.matrix(read.csv("level_6_Taxonomy_P.csv", row.names=1))

# Prepare files for SPIEC-EASI
OTU.6.P = otu_table(otumat.6.P, taxa_are_rows = TRUE)
TAX.6.P = tax_table(taxmat.6.P)
abund.t.6.P <- t(OTU.6.P)
genus.6.P <- TAX.6.P[,6]
physeq.6.P = phyloseq(OTU.6.P, TAX.6.P, sampledata)

# Perform SPIEC-EASI
se.6.P <- spiec.easi(physeq.6.P, method='mb', lambda.min.ratio=1e-2, nlambda=20,
pulsar.params=list(rep.num=50))
net1.6.P <- adj2igraph(getRefit(se.6.P),  vertex.attr=list(name=taxa_names(physeq.6.P)))

# Adjust network layout
am.coord.6.P <- layout.fruchterman.reingold(net1.6.P)
net2.6.P <- adj2igraph(getRefit(se.6.P))
vsize.6.P <- rowMeans(clr(abund.t.6.P, 1))+10
write.csv(vsize.6.P,"vsize_6_P.csv", row.names = TRUE)
optbeta.6.P <- as.matrix(symBeta(getOptBeta(se.6.P)))
edge_cols.6.P <-  ifelse(optbeta.6.P>0, 'red', 'blue')[upper.tri(optbeta.6.P) &
optbeta.6.P!=0]
E(net2.6.P)$color=edge_cols.6.P
weights.6.P <- optbeta.6.P[upper.tri(optbeta.6.P) & optbeta.6.P!=0]*20+1
weights.6.P <- abs(weights.6.P)
E(net2.6.P)$weight <- weights.6.P
V(net2.6.P)$name <- genus.6.P

# Plot initial network (contains all connections)
plot(net2.6.P, layout = am.coord.6.P, vertex.size = vsize.6.P, edge.width =
E(net2.6.P)$weight, vertex.label.cex = 0.5, vertex.label.color = "black")
net2df.6.P <- get.data.frame(net2.6.P)
write.csv(net2df.6.P,"net1df_6_P.csv", row.names = FALSE)
#Manually remove any rows without Kazachstania slooffiae
#Edit vertex size file (vsize.csv) to contain only taxa and Kazachstania slooffiae in the
net2df file after removing rows without the fungus

# Import edited csv file containing only Kazachstania slooffiae connections
KS.df.6.P <-(read.csv("net1df_KS_6_P.csv"))
net3.6.P <- graph_from_data_frame(KS.df.6.P)

# Color vertices: bacteria green and fungi blue
V(net3.6.P)$color <- "seagreen1"
```

```
V(net3.6.P)$color[1] <- "skyblue1"

# Import file for vertex sizes
vsize.KS.6.P <- read.csv("vsize_KS_6_P.csv")

# Adjust layout of network
am.coord.KS.6.P <- layout.fruchterman.reingold(net3.6.P)
plot(net3.6.P,layout = am.coord.KS.6.P, vertex.size = vsize.KS.6.P$x, vertex.label.cex =
0.5, vertex.label.color = "black", edge.arrow.size=0.05, edge.width = KS.df.6.P$weight)

# Obtain stability
getStability(se.6.P)
# 0.03625496
```

Nursery

```
# Load additional files for nursery
otumat.6.N <-(read.csv("level_6_with_KS_PCR_N.csv", row.names=1))
taxmat.6.N <- as.matrix(read.csv("level_6_Taxonomy_N.csv", row.names=1))

# Prepare files for SPIEC-EASI
OTU.6.N = otu_table(otumat.6.N, taxa_are_rows = TRUE)
TAX.6.N = tax_table(taxmat.6.N)
abund.t.6.N <- t(OTU.6.N)
genus.6.N <- TAX.6.N[,6]
physeq.6.N = phyloseq(OTU.6.N, TAX.6.N, sampledata)

# Perform SPIEC-EASI
se.6.N <- spiec.easi(physeq.6.N, method='mb', lambda.min.ratio=1e-2, nlambda=20,
pulsar.params=list(rep.num=50))
net1.6.N <- adj2igraph(getRefit(se.6.N),  vertex.attr=list(name=taxa_names(physeq.6.N)))

# Adjust network layout
am.coord.6.N <- layout.fruchterman.reingold(net1.6.N)
net2.6.N <- adj2igraph(getRefit(se.6.N))
vsize.6.N <- rowMeans(clr(abund.t.6.N, 1))+10
write.csv(vsize.6.N,"vsize_6_N.csv", row.names = TRUE)
optbeta.6.N <- as.matrix(symBeta(getOptBeta(se.6.N)))
edge_cols.6.N <-  ifelse(optbeta.6.N>0, 'red', 'blue')[upper.tri(optbeta.6.N) &
optbeta.6.N!=0]
E(net2.6.N)$color=edge_cols.6.N
weights.6.N <- optbeta.6.N[upper.tri(optbeta.6.N) & optbeta.6.N!=0]*20+1
weights.6.N <- abs(weights.6.N)
E(net2.6.N)$weight <- weights.6.N
V(net2.6.N)$name <- genus.6.N

# Plot initial network (contains all connections)
plot(net2.6.N, layout = am.coord.6.N, vertex.size = vsize.6.N, edge.width =
E(net2.6.N)$weight, vertex.label.cex = 0.5, vertex.label.color = "black")
net2df.6.N <- get.data.frame(net2.6.N)
write.csv(net2df.6.N,"net1df_6_N.csv", row.names = FALSE)
#Manually remove any rows without Kazachstania slooffiae
#Edit vertex size file (vsize.csv) to contain only taxa and Kazachstania slooffiae in the
net2df file after removing rows without the fungus

# Import edited csv file containing only Kazachstania slooffiae connections
KS.df.6.N <-(read.csv("net1df_KS_6_N.csv"))
net3.6.N <- graph_from_data_frame(KS.df.6.N)


# Color vertices: bacteria green and fungi blue
```

```
V(net3.6.N)$color <- "seagreen1"
V(net3.6.N)$color[1] <- "skyblue1"

# Import file for vertex sizes
vsize.KS.6.N <- read.csv("vsize_KS_6_N.csv")

# Adjust layout of network
am.coord.KS.6.N <- layout.fruchterman.reingold(net3.6.N)
plot(net3.6.N,layout = am.coord.KS.6.N, vertex.size = vsize.KS.6.N$x, vertex.label.cex =
0.5, vertex.label.color = "black", edge.arrow.size=0.05, edge.width =KS.df.6.N$weight)

# Obtain stability
getStability(se.6.N)
#0.04480403
```

Growth Adult

```
# Load additional files for growth adult
otumat.6.G <-(read.csv("level_6_with_KS_PCR_G.csv", row.names=1))
taxmat.6.G <- as.matrix(read.csv("level_6_Taxonomy_G.csv", row.names=1))

# Prepare files for SPIEC-EASI
OTU.6.G = otu_table(otumat.6.G, taxa_are_rows = TRUE)
TAX.6.G = tax_table(taxmat.6.G)
abund.t.6.G <- t(OTU.6.G)
genus.6.G <- TAX.6.G[,6]
physeq.6.G = phyloseq(OTU.6.G, TAX.6.G, sampledata)

#Perform SPIEC-EASI
se.6.G <- spiec.easi(physeq.6.G, method='mb', lambda.min.ratio=1e-2, nlambda=20,
pulsar.params=list(rep.num=50))
net1.6.G <- adj2igraph(getRefit(se.6.G),  vertex.attr=list(name=taxa_names(physeq.6.G)))

#Adjust network layout
am.coord.6.G <- layout.fruchterman.reingold(net1.6.G)
net2.6.G <- adj2igraph(getRefit(se.6.G))
vsize.6.G <- rowMeans(clr(abund.t.6.G, 1))+10
write.csv(vsize.6.G,"vsize_6_G.csv", row.names = TRUE)
optbeta.6.G <- as.matrix(symBeta(getOptBeta(se.6.G)))
edge_cols.6.G <-  ifelse(optbeta.6.G>0, 'red', 'blue')[upper.tri(optbeta.6.G) &
optbeta.6.G!=0]
E(net2.6.G)$color=edge_cols.6.G
weights.6.G <- optbeta.6.G[upper.tri(optbeta.6.G) & optbeta.6.G!=0]*20+1
weights.6.G <- abs(weights.6.G)
E(net2.6.G)$weight <- weights.6.G
V(net2.6.G)$name <- genus.6.G

#Plot initial network (contains all connections)
plot(net2.6.G, layout = am.coord.6.G, vertex.size = vsize.6.G, edge.width =
E(net2.6.G)$weight, vertex.label.cex = 0.5, vertex.label.color = "black")
net2df.6.G <- get.data.frame(net2.6.G)
write.csv(net2df.6.G,"net1df_6_G.csv", row.names = FALSE)
#Manually remove any rows without Kazachstania slooffiae
#Edit vertex size file (vsize.csv) to contain only taxa and Kazachstania slooffiae in the
net2df file after removing rows without the fungus

# Import edited csv file containing only Kazachstania slooffiae connections
KS.df.6.G <-(read.csv("net1df_KS_6_G.csv"))
net3.6.G <- graph_from_data_frame(KS.df.6.G)


# Color vertices: bacteria green and fungi blue
```

```
V(net3.6.G)$color <- "seagreen1"
V(net3.6.G)$color[1] <- "skyblue1"


# Import file for vertex sizes
vsize.KS.6.G <- read.csv("vsize_KS_6_G.csv")

# Adjust layout of network
am.coord.KS.6.G <- layout.fruchterman.reingold(net3.6.G)
plot(net3.6.G,layout = am.coord.KS.6.G, vertex.size = vsize.KS.6.G$x, vertex.label.cex =
0.5, vertex.label.color = "black", edge.arrow.size=0.05,  edge.width=KS.df.6.G$weight)

# Obtain stability
getStability(se.6.G)
#0.03548979
```

```
# Close RStudio
```
