# Supplementary figures and images for: Stability and volatility shape the gut bacteriome and Kazachstania slooffiae dynamics in preweaning, nursery and adult pigs
Source: Sci Rep. 2022 Sep 5;12:15080. doi: 10.1038/s41598-022-19093-9 (PMC9445069; doi:10.1038/s41598-022-19093-9)

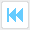

Supplement: Supplementary file 3 — Supplementary Information 3. [file 41598_2022_19093_MOESM3_ESM.qzv › a21c9230-1107-429d-8617-7e623d9ddfe3/data/img/reset.png]

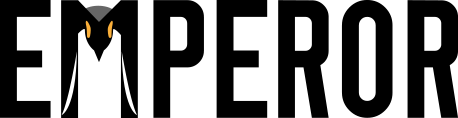

Supplement: Supplementary file 3 — Supplementary Information 3. [file 41598_2022_19093_MOESM3_ESM.qzv › a21c9230-1107-429d-8617-7e623d9ddfe3/data/img/emperor.png]

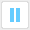

Supplement: Supplementary file 3 — Supplementary Information 3. [file 41598_2022_19093_MOESM3_ESM.qzv › a21c9230-1107-429d-8617-7e623d9ddfe3/data/img/pause.png]

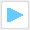

Supplement: Supplementary file 3 — Supplementary Information 3. [file 41598_2022_19093_MOESM3_ESM.qzv › a21c9230-1107-429d-8617-7e623d9ddfe3/data/img/play.png]

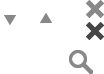

Supplement: Supplementary file 3 — Supplementary Information 3. [file 41598_2022_19093_MOESM3_ESM.qzv › a21c9230-1107-429d-8617-7e623d9ddfe3/data/vendor/css/chosen-sprite@2x.png]

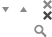

Supplement: Supplementary file 3 — Supplementary Information 3. [file 41598_2022_19093_MOESM3_ESM.qzv › a21c9230-1107-429d-8617-7e623d9ddfe3/data/vendor/css/chosen-sprite.png]

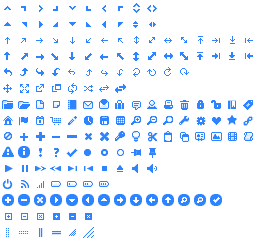

Supplement: Supplementary file 3 — Supplementary Information 3. [file 41598_2022_19093_MOESM3_ESM.qzv › a21c9230-1107-429d-8617-7e623d9ddfe3/data/vendor/css/images/ui-icons_2e83ff_256x240.png]

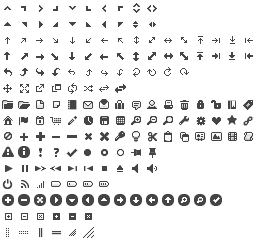

Supplement: Supplementary file 3 — Supplementary Information 3. [file 41598_2022_19093_MOESM3_ESM.qzv › a21c9230-1107-429d-8617-7e623d9ddfe3/data/vendor/css/images/ui-icons_454545_256x240.png]

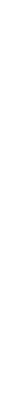

Supplement: Supplementary file 3 — Supplementary Information 3. [file 41598_2022_19093_MOESM3_ESM.qzv › a21c9230-1107-429d-8617-7e623d9ddfe3/data/vendor/css/images/ui-bg_glass_65_ffffff_1x400.png]

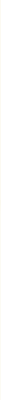

Supplement: Supplementary file 3 — Supplementary Information 3. [file 41598_2022_19093_MOESM3_ESM.qzv › a21c9230-1107-429d-8617-7e623d9ddfe3/data/vendor/css/images/ui-bg_glass_55_fbf9ee_1x400.png]

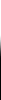

Supplement: Supplementary file 3 — Supplementary Information 3. [file 41598_2022_19093_MOESM3_ESM.qzv › a21c9230-1107-429d-8617-7e623d9ddfe3/data/vendor/css/images/ui-bg_highlight-soft_75_cccccc_1x100.png]

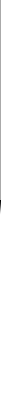

Supplement: Supplementary file 3 — Supplementary Information 3. [file 41598_2022_19093_MOESM3_ESM.qzv › a21c9230-1107-429d-8617-7e623d9ddfe3/data/vendor/css/images/ui-bg_glass_75_e6e6e6_1x400.png]

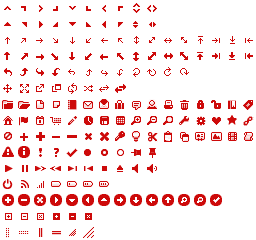

Supplement: Supplementary file 3 — Supplementary Information 3. [file 41598_2022_19093_MOESM3_ESM.qzv › a21c9230-1107-429d-8617-7e623d9ddfe3/data/vendor/css/images/ui-icons_cd0a0a_256x240.png]

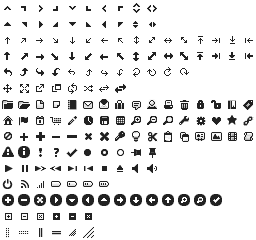

Supplement: Supplementary file 3 — Supplementary Information 3. [file 41598_2022_19093_MOESM3_ESM.qzv › a21c9230-1107-429d-8617-7e623d9ddfe3/data/vendor/css/images/ui-icons_222222_256x240.png]

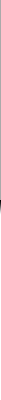

Supplement: Supplementary file 3 — Supplementary Information 3. [file 41598_2022_19093_MOESM3_ESM.qzv › a21c9230-1107-429d-8617-7e623d9ddfe3/data/vendor/css/images/ui-bg_glass_75_dadada_1x400.png]

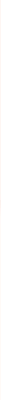

Supplement: Supplementary file 3 — Supplementary Information 3. [file 41598_2022_19093_MOESM3_ESM.qzv › a21c9230-1107-429d-8617-7e623d9ddfe3/data/vendor/css/images/ui-bg_glass_95_fef1ec_1x400.png]

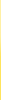

Supplement: Supplementary file 3 — Supplementary Information 3. [file 41598_2022_19093_MOESM3_ESM.qzv › a21c9230-1107-429d-8617-7e623d9ddfe3/data/vendor/css/images/ui-bg_highlight-soft_75_ffe45c_1x100.png]

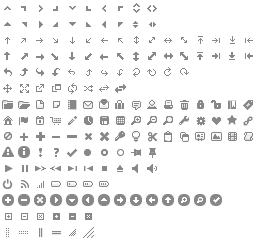

Supplement: Supplementary file 3 — Supplementary Information 3. [file 41598_2022_19093_MOESM3_ESM.qzv › a21c9230-1107-429d-8617-7e623d9ddfe3/data/vendor/css/images/ui-icons_888888_256x240.png]

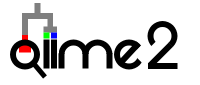

Supplement: Supplementary file 3 — Supplementary Information 3. [file 41598_2022_19093_MOESM3_ESM.qzv › a21c9230-1107-429d-8617-7e623d9ddfe3/data/q2templateassets/img/qiime2-rect-200.png]
