# Supplementary material for: Stability and volatility shape the gut bacteriome and Kazachstania slooffiae dynamics in preweaning, nursery and adult pigs
Source: Sci Rep. 2022 Sep 5;12:15080. doi: 10.1038/s41598-022-19093-9 (PMC9445069; doi:10.1038/s41598-022-19093-9)
Supplement: Supplementary file 4 — Supplementary Information 4. [file 41598_2022_19093_MOESM4_ESM.qzv › 40103518-09bc-431a-96fb-58964e2d3fe1/data/index.html]

q2\_diversity : alpha\_group\_significance


The following metadata columns have been omitted because they didn't
contain categorical data:
**Age**

The following categorical metadata columns have been omitted because the
number of groups was equal to the number of samples, there was only a
single group, or the column consisted only of missing data:
**DNA-ID, Description, Farm, Inside\_or\_Outside, SampleType**

Download raw data as TSV

---

## Kruskal-Wallis (all groups)

|  | Result |
| --- | --- |
| H |  |
| p-value |  |

---

## Kruskal-Wallis (pairwise)

Download CSV
