# Supplementary material for: Stability and volatility shape the gut bacteriome and Kazachstania slooffiae dynamics in preweaning, nursery and adult pigs
Source: Sci Rep. 2022 Sep 5;12:15080. doi: 10.1038/s41598-022-19093-9 (PMC9445069; doi:10.1038/s41598-022-19093-9)
Supplement: Supplementary file 7 — Supplementary Information 7. [file 41598_2022_19093_MOESM7_ESM.qzv › 6f17b349-ef2e-4cb9-9341-c9bf26230f05/data/index.html]

q2-longitudinal : volatility


### Volatility Control Chart

### Plot Controls

Click the individual group value labels in the legend to toggle
their visibility in the displayed plot.

---
